# Auraptene Enhances AMP-Activated Protein Kinase Phosphorylation and Thereby Inhibits the Proliferation, Migration and Expression of Androgen Receptors and Prostate-Specific Antigens in Prostate Cancer Cells

**DOI:** 10.3390/ijms242116011

**Published:** 2023-11-06

**Authors:** Yasuyuki Akasaka, Shun Hasei, Yukino Ohata, Machi Kanna, Yusuke Nakatsu, Hideyuki Sakoda, Midori Fujishiro, Akifumi Kushiyama, Hiraku Ono, Akio Matsubara, Nobuyuki Hinata, Tomoichiro Asano, Takeshi Yamamotoya

**Affiliations:** 1 Department of Biomedical Chemistry, Graduate School of Biomedical and Health Sciences, Hiroshima University, Hiroshima 734-8553, Japan; 2 Department of Bioregulatory Sciences, Faculty of Medicine, University of Miyazaki, Miyazaki 889-1692, Japan; 3 Division of Diabetes and Metabolic Diseases, Nihon University School of Medicine, Tokyo 173-8610, Japan; 4 Department of Pharmacotherapy, Meiji Pharmaceutical University, Kiyose 204-8588, Japan; 5 Department of Clinical Cell Biology, Graduate School of Medicine, Chiba University, Chiba 260-8670, Japan; 6 Department of Urology, JA Hiroshima General Hospital, Hatsukaichi 738-8503, Japan; 7 Department of Urology, Graduate School of Biomedical and Health Sciences, Hiroshima University, Hiroshima 734-8553, Japan

**Keywords:** auraptene, AMPK, LKB1, prostate cancer, androgen receptor, prostate-specific antigen

## Abstract

*Citrus hassaku* extract reportedly activates AMPK. Because this extract contains an abundance of auraptene, we investigated whether pure auraptene activates AMPK and inhibits proliferation using prostate cancer cell lines. Indeed, auraptene inhibited the proliferation and migration of LNCaP cells and induced phosphorylation of AMPK or its downstream ACC in LNCaP, PC3, and HEK-293 cells, but not in DU145 cells not expressing LKB1. In addition, the mTOR-S6K pathway, located downstream from activated AMPK, was also markedly suppressed by auraptene treatment. Importantly, it was shown that auraptene reduced androgen receptor (AR) and prostate-specific antigen (PSA) expressions at both the protein and the mRNA level. This auraptene-induced downregulation of PSA was partially but significantly reversed by treatment with AMPK siRNA or the AMPK inhibitor compound C, suggesting AMPK activation to, at least partially, be causative. Finally, in DU145 cells lacking the LKB1 gene, exogenously induced LKB1 expression restored AMPK phosphorylation by auraptene, indicating the essential role of LKB1. In summary, auraptene is a potent AMPK activator that acts by elevating the AMP/ATP ratio, thereby potentially suppressing prostate cancer progression, via at least three molecular mechanisms, including suppression of the mTOR-S6K pathway, reduced lipid synthesis, and AR downregulation caused by AMPK activation.

## 1. Introduction

The incidence of cancer has steadily been rising worldwide with rapid population aging. Prostate cancer is one of the most common cancers in men, with 1.41 million new cases in 2020 [1]. While several treatment options are available, including surgery, radiation, and anti-androgen therapy, another approach is expectant management, in which cancer progression is monitored without definitive therapy, reflecting how slow-growing prostate tumors tend to be [2,3,4]. Compared with the side effects of widely used cancer drugs, certain natural products with low toxicity levels might be beneficial and merit consideration for treatment and/or prevention of cancer development.

AMP-activated protein kinase (AMPK) is a serine/threonine kinase governing various cellular processes, including metabolism, mitochondrial biogenesis, autophagy, cell growth, and proliferation [5,6]. AMPK is activated in response to cellular energy depletion (i.e., increased intracellular AMP/ATP ratio) by LKB1-mediated phosphorylation of Thr172 in the AMPKα subunit, which can also be mediated by CAMKK2 when calcium flux occurs. AMPK activation has been shown to suppress the mammalian target of the rapamycin complex 1 (mTORC1) pathway by directly phosphorylating TSC2 and RAPTOR [7,8,9,10,11]. AMPK also activates p53 by phosphorylating Ser15 (in humans) in p53, thereby promoting arrest of the cell cycle [12,13]. Therefore, activating AMPK has the potential to inhibit cancer proliferation via the inhibition of mTORC1 and the activation of p53. Natural products activating AMPK might thus exert beneficial effects when used in combination with currently available therapies and might also be applicable to patients diagnosed with prostate cancer but still not eligible for definitive therapy.

Auraptene, also known as 7-geranyloxycoumarin, is a member of the coumarin family of compounds that can be isolated from citrus fruits such as *Citrus aurantium* (bitter orange), *Citrus hassaku*, and grapefruit. It has been extensively studied in terms of its therapeutic potential against cancer as well as for exerting other actions such as anti-inflammatory or neuroprotective effects [14,15,16,17]. Tang et al. reported that dietary auraptene effectively reduced high-grade lesions of the prostate in TRAP rats, a transgenic rat model developing adenocarcinomas of the prostate that express the SV40 T antigen transgene under control of the probasin promoter in vivo, and that auraptene consistently inhibited the proliferation of human prostate cancer cell lines, including LNCaP, DU145, and PC3, in vitro [18]. Lee et al. also demonstrated that auraptene increased apoptosis in DU145 and PC3 cell lines [19]. However, the precise mechanisms by which auraptene inhibits proliferation and increases apoptosis in prostate cancer cells are yet to be identified. It was recently reported that *Citrus hassaku* extract powder (CHEP) upregulated PGC-1α and increased the mitochondrial content of muscles in mice fed a CHEP-containing diet by promoting SIRT3 expression and AMPK activation [20].

Based on the aforementioned prior reports and the evidence that CHEP contains an abundance of auraptene, we speculated that auraptene activates AMPK. We thus performed experiments using multiple cell lines, including LNCaP, DU145, PC3, and HEK-293. Indeed, we recently reported significant AMPK-activating and anti-proliferative effects of carnosic acid and carnosol, both of which are components of rosemary extracts, based on our results obtained by screening hundreds of commercially available extracts derived from natural products [21]. In this study, we demonstrated AMPK activation by auraptene and its inhibitory effects on cell proliferation and migration. The inhibitory effect of auraptene on cell growth as well as reduced expression and activity of the androgen receptor (AR), a key driver of prostate cancer progression, raise the possibility of auraptene, a major component of *Citrus hassaku,* being useful as a treatment for or protection against the development of prostate cancer.

## 2. Results

### 2.1. Auraptene Suppresses Proliferation and Migration of Prostate Cancer LNCaP Cells

**Figure 1:**
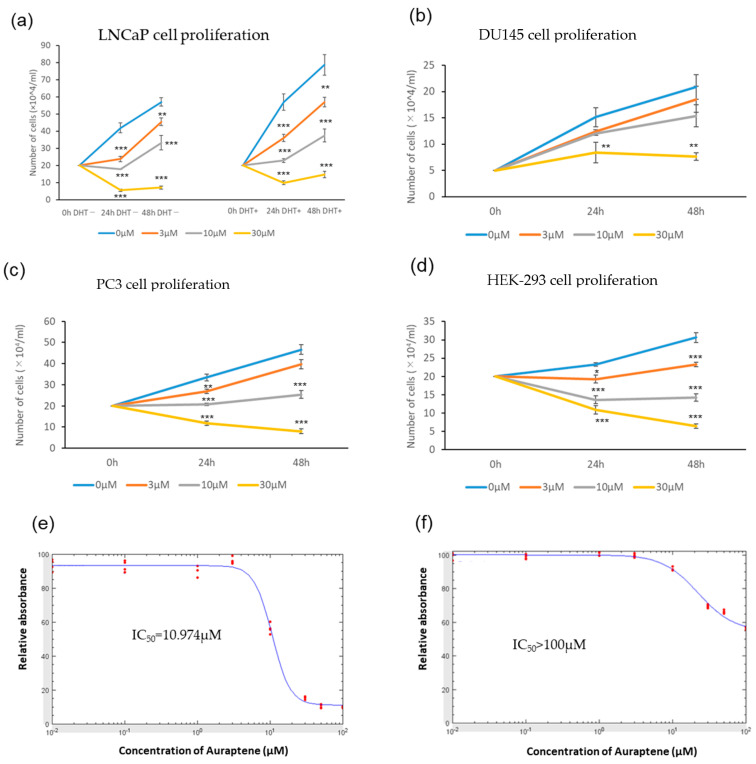

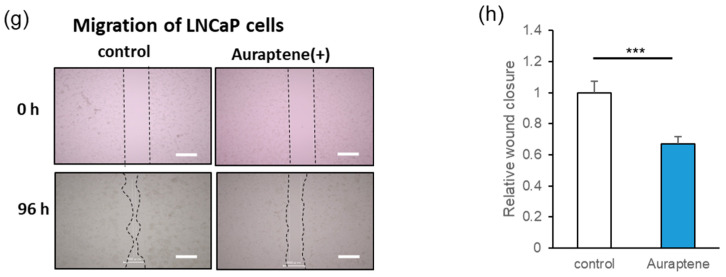
Auraptene suppresses the proliferation and migration of prostate cancer LNCaP cells. (**a**) The effect of auraptene on the proliferation of LNCaP cells. Cells were treated with auraptene for 0, 24, or 48 h at the indicated concentrations (with or without DHT (10 nM)) (*n* = 4). (**b**–**d**) The effect of auraptene on the proliferation of (**b**) DU145, (**c**) PC3, and (**d**) HEK-293 cells (without DHT) (*n* = 4). (**e**,**f**) Determination of the IC_50_ of auraptene against LNCaP and DU145 cells using CCK-8 assay. Cells were treated with auraptene for 24 h at the indicated concentrations, and then absorbances at 450 nm were measured. Absorbances relative to auraptene-untreated cells (red dots, *n* = 4) were used to calculate the IC_50_ (blue lines: fitting curves). (**g**,**h**) Wound healing assay of LNCaP cells. Migration distances were measured under a microscope before and 96 h after administration of 30 μM auraptene (or vehicle as a control). Representative images (scale bar: 500 μm) (**g**) and quantification of wound closure (relative to control) (*n* = 4) (**h**). (* *p* < 0.05, ** *p* < 0.01, *** *p* < 0.001).

First, to investigate the effects of auraptene on the growth of prostate cancer LNCaP, DU145, PC3, and HEK-293 cells, equal numbers of these cell types were separately seeded and then treated with various concentrations (0, 3, 10, 30 μM) of auraptene for 0 h, 24 h, or 48 h, followed by counting cell numbers to measure cellular proliferation. Auraptene was shown to inhibit cell growth, irrespective of the presence or absence of dihydrotestosterone (DHT), in a concentration-dependent manner at concentrations no lower than 3 μM (left and right panels of Figure 1a). In contrast, the proliferation of DU145 cells was not significantly suppressed at either 3 or 10 μM of auraptene, while significant suppression was observed at 30 μM (Figure 1b). Similar suppressive effects of auraptene were observed in PC3 and HEK-293 cells as well as in LNCaP cells (Figure 1c,d). Thus, the DU145 cell line appeared to be significantly more resistant to the growth-inhibitory effects exerted by auraptene.

We also performed the CCK-8 assay, which reflects both cell proliferation and viability. The IC_50_ of auraptene against LNCaP cells and DU145 cells, calculated from the CCK-8 assay, were 11.0 μM and above 100 μM, respectively (Figure 1e,f), much larger in DU145 cells than in LNCaP cells, which is consistent with the cell counting assay results. (Figure 1a,b). The results of CCK-8 assays in PC3 and HEK-293 cells, as shown in Supplementary Figure S1, were also in line with the results presented in Figure 1c,d.

Next, we performed a wound-healing assay to examine the effects of auraptene on the migration ability using LNCaP cells. In good agreement with the results of the cellular proliferation assays, the wound-healing assay revealed significant attenuation of cell migration in response to treatment with auraptene at the 30 μM concentration (Figure 1g,h). These observations suggest that auraptene suppresses cancer cell proliferation and migration.

### 2.2. Auraptene Induces AMPK Activation

LNCaP, DU145, PC3, and HEK-293 cells were incubated with DMSO alone or in addition to various concentrations of auraptene (3, 10, and 30 μM) or 5-aminoimidazole-4-carboxamide 1-β-D-ribofuranoside (AICAR), a positive control for AMPK activation, for 8 h. We then evaluated phosphorylation levels of AMPKα Thr172, which is essential for its activation, and its downstream target acetyl Co-A carboxylase (ACC) Ser79 phosphorylation.

**Figure 2:**
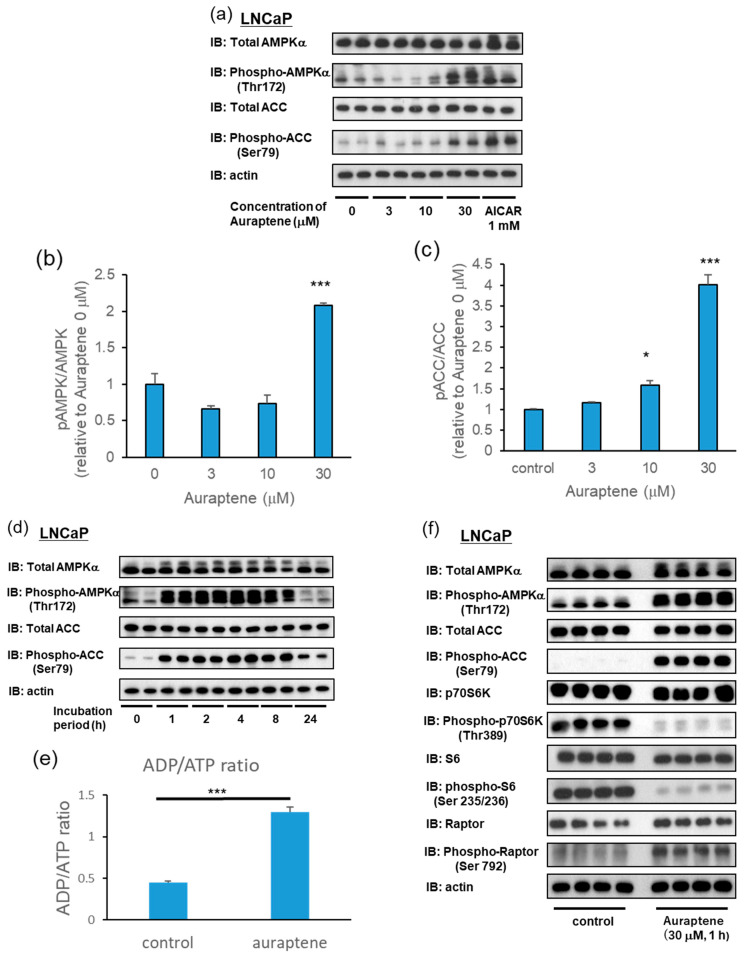
Auraptene induces AMPK activation in LNCaP cells. (**a**–**c**) LNCaP cells were treated with auraptene at the indicated concentrations for 8 h. AICAR (1 mM) was employed as a positive control. (**a**) AMPKα, phospho-AMPKα, ACC, phospho-ACC, and actin in LNCaP cells were detected using immunoblotting. (**b**,**c**) Quantification of relative band intensities (pAMPK/AMPK (**b**) and pACC/ACC (**c**)) (*n* = 4). (**d**) Western blot analysis. LNCaP cells were treated with 30 μM auraptene for 1, 2, 4, 8, or 24 h. (**e**) LNCaP cells were treated with 30 μM auraptene for 1 h, and the intracellular ADP/ATP ratio was determined using an ADP/ATP Ratio Assay Kit (*n* = 4). (**f**) Western blot analysis. LNCaP cells were treated with 30 μM auraptene for 1 h. (* *p* < 0.05, *** *p* < 0.001).

In LNCaP cells, auraptene induced significant AMPK phosphorylation at 30 μM, along with increased downstream ACC phosphorylation, which was statistically significant even at 10 μM (Figure 2a–c). We next performed time-course examinations. LNCaP cells were incubated with auraptene at 30 μM for various times (1, 2, 4, 8, and 24 h). Marked AMPK and ACC phosphorylations were observed as early as 1 h after administration and persisted up to 8 h in LNCaP cells (Figure 2d).

The total AMPK and total ACC levels remained essentially unchanged. Phosphorylation of AMPK at Thr172 is mediated by one of two upstream kinases, either LKB1 or CAMKK2 [6,22,23]. LKB1 activates AMPK in response to energy depletion (i.e., increases in the intracellular AMP/ATP and ADP/ATP ratios), whereas CAMKK2 activates AMPK in response to elevated calcium. Auraptene increased the cellular ADP/ATP ratio (Figure 2e), suggesting that auraptene activated AMPK via its impacts on cellular ATP production.

AMPK is known to be an upstream regulator of mTOR, which supports cell proliferation via activation of the p70S6K signaling pathway. Activated AMPK inhibits mTORC1 by phosphorylating its upstream inhibitor TSC2 [7] as well as directly phosphorylating its Raptor component [8]. As illustrated in Figure 2f, auraptene markedly attenuates the phosphorylation of p70S6K and its downstream target S6. These results suggest that auraptene suppresses the growth of LNCaP cells, at least in part, by inhibiting the mTOR-S6K signaling pathway.

**Figure 3:**
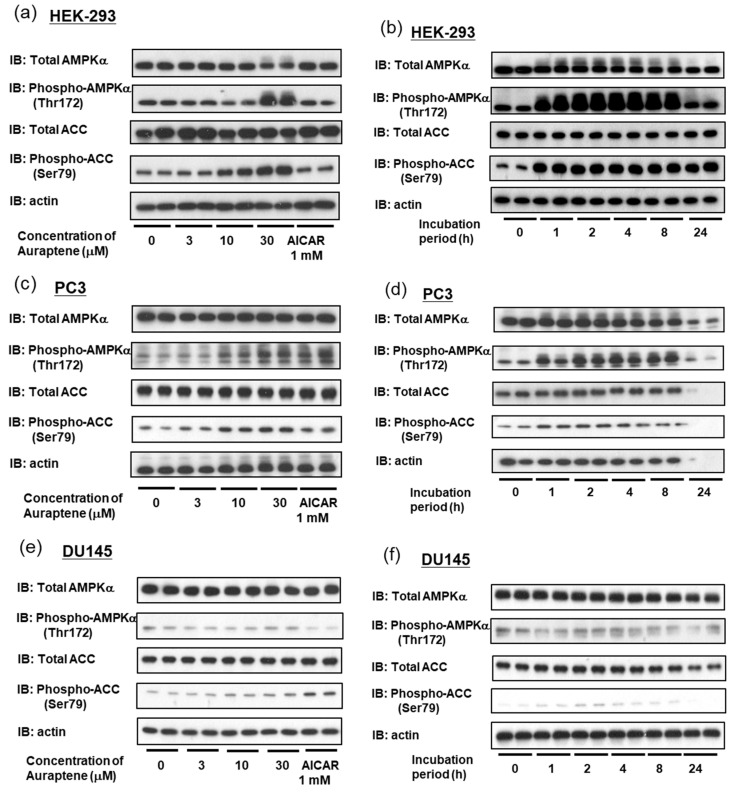
Auraptene induces AMPK activation in HEK-293 and PC3 cells but not in DU145 cells. (**a**,**c**,**e**) AMPKα, phosphor-AMPKα, ACC, phosphor-ACC, and actin were detected using immunoblotting after treatment with auraptene at the indicated concentrations for 8 h. AICAR (1 mM) was employed as a positive control. (**a**) HEK-293, (**c**) PC3, and (**e**) DU145. (**b**,**d**,**f**) Cells were treated with 30 μM auraptene for 1, 2, 4, 8, or 24 h, and immunoblotting was then performed. (**b**) HEK-293, (**d**) PC3, and (**f**) DU145.

Auraptene-induced phosphorylations of AMPK and ACC were also observed in HEK-293 (Figure 3a,b) and PC3 (Figure 3c,d) cells. The concentration-dependency and time-course profiles of auraptene using the HEK-293 and PC3 cell lines were similar to those obtained with LNCaP cells. Interestingly, however, auraptene failed to activate AMPK or subsequent ACC phosphorylation in DU145 cells (Figure 3e,f).

### 2.3. Auraptene Attenuates the Expressions of AR and PSA in LNCaP Cells

The AR signaling pathway is a key driver of prostate cancer progression, such that inhibiting the AR signaling pathway is of major importance in the treatment of this malignancy. Several prior reports have raised the possibility that AMPK activating agents, such as metformin, downregulate AR activity and the expressions of its target genes [24,25,26].

**Figure 4:**
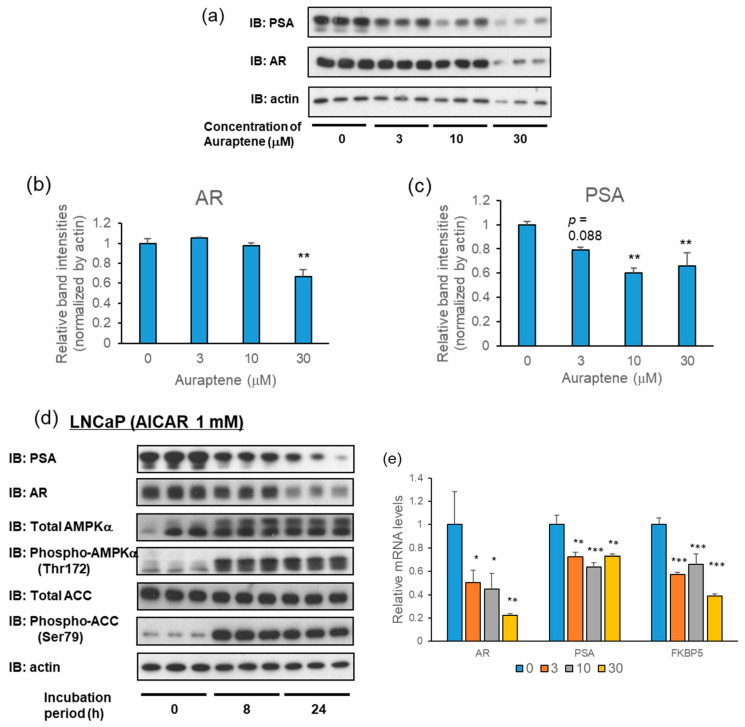
Auraptene attenuates the expressions of AR and PSA in LNCaP cells. (**a**) Protein levels of AR and PSA in LNCaP cells were examined using immunoblotting after treatment with 0, 3, 10, or 30 μM auraptene for 24 h. (**b**,**c**) Relative band intensities of (**b**) AR and (**c**) PSA protein levels are shown as bar graphs (*n* = 3). (**d**) Immunoblotting analysis of AR and PSA in LNCaP cells after treatment with 1 mM AICAR for 8 or 24 h. (**e**) LNCaP cells were treated with auraptene at 30 μM for 6 h, and the mRNAs of AR, PSA, and FKBP5 were examined using quantitative real-time PCR analysis. (*n* = 4–6). (* *p* < 0.05, ** *p* < 0.01, *** *p* < 0.001).

Therefore, employing Western blot analysis, we investigated the effects of auraptene on the expressions of AR and its target gene PSA. LNCaP cells were treated with various concentrations of auraptene (3, 10, and 30 μM) for 24 h. AR and PSA protein levels were significantly decreased at 30 μM and at 10 and 30 μM, respectively, after the administration of auraptene (Figure 4a–c). Similar effects were observed when the 1 mM concentration of AICAR was employed for stimulation (Figure 4d).

In addition to the protein level, the effects of auraptene on the levels of AR and PSA mRNA were investigated using a quantitative real-time PCR analysis. When LNCaP cells were treated with 30 μM auraptene for 6 h, the levels of AR and PSA mRNA, as well as that of another AR target gene, FKBP5, were significantly decreased (Figure 4e). These observations are consistent with the decreases in AR and PSA protein levels. Our results suggest that, in LNCaP cells, auraptene-induced reductions in AR and PSA proteins are attributable to the suppression of their mRNA expression levels.

### 2.4. Suppression of AR Target Gene PSA by Auraptene in LNCaP Cells Is at Least Partially Mediated via AMPK Activation

**Figure 5:**
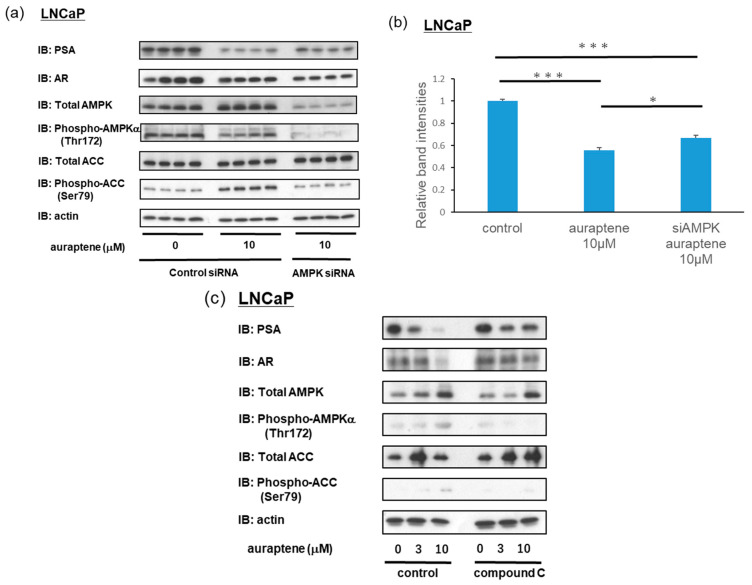
Auraptene-induced downregulation of PSA was partially reversed by suppressing AMPK. (**a**) Effects of the treatment with AMPK siRNA on the protein levels of AR and PSA. LNCaP cells were transfected with control siRNA or AMPK siRNA for 72 h and then treated with 10 μM of auraptene (or vehicle as a control) for 24 h. PSA, AR, AMPK, phosphor-AMPKα, ACC, phosphor-ACC, and actin were detected using immunoblotting. (**b**) Quantification of relative band intensities (PSA/actin) (*n* = 4). (**c**) Effects of compound C on the protein levels of AR and PSA. LNCaP cells were pre-treated with 5 μM of compound C for 1 h and then treated with 0, 3, or 10 μM of auraptene for 24 h. (* *p* < 0.05, *** *p* < 0.001).

Next, we investigated whether the downregulations of AR and PSA by auraptene were dependent on the ability of auraptene to activate AMPK. LNCaP cells were transfected with control siRNA or AMPK siRNA for 72 h and then treated with either the vehicle or auraptene at a 10 μM concentration for 24 h. The analysis conducted 24 h after auraptene administration revealed that AMPK phosphorylation had already subsided while ACC phosphorylation remained upregulated (Figure 5a). As expected, in cells treated with AMPK siRNA, auraptene-induced phosphorylation of ACC showed marked attenuation (Figure 5a). Moreover, auraptene-induced reductions in PSA protein levels were slightly but significantly reversed when the cells were transfected with AMPK siRNA, as compared to those transfected with control siRNA (Figure 5a,b). Similar results were confirmed in experiments using the AMPK inhibitor compound C (Figure 5c). LNCaP cells were pre-treated with 5 μM compound C for 1 h, and then treated with either the vehicle or auraptene at a concentration of 3 or 10 μM for 24 h. Auraptene-induced reductions in the PSA and AR protein levels were partially reversed when LNCaP cells were pretreated with compound C. Collectively, these results indicate that auraptene-induced downregulations of PSA expressions were mediated by AMPK activation.

### 2.5. Auraptene Induces AMPK Activation through the LKB1 Pathway

**Figure 6:**
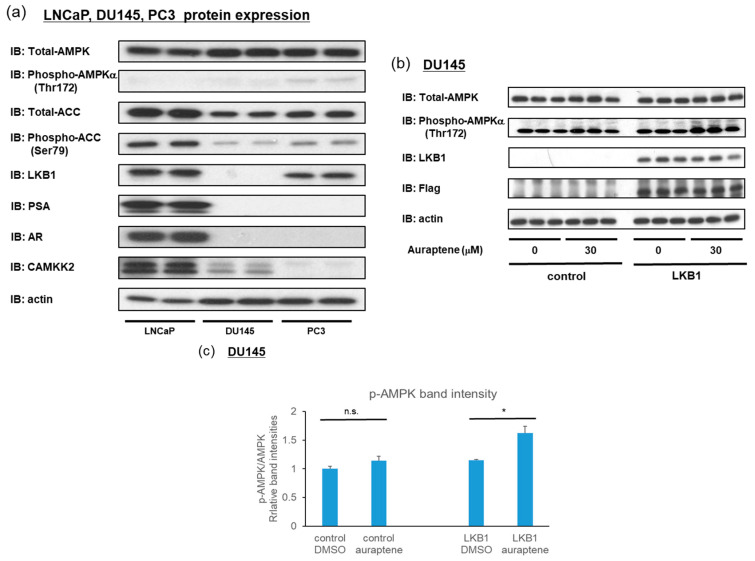
LKB1 is essential for auraptene to activate AMPK. (**a**) The protein expression profiles of AMPKα, phospho-AMPKα, ACC, phospho-ACC, LKB1, PSA, AR, CAMKK2, and actin were compared among LNCaP, DU145, and PC3 cells using immunoblotting. Notably, DU145 cells lack LKB1. (**b**) Effect of introduction of the LKB1 expression plasmid into DU145 cells lacking LKB1 on AMPKα phosphorylation. DU145 cells were transfected with pcDNA3.1(-) or MTF (myc-TEV-Flag-tagged)-LKB1 plasmids for 48 h, and then treated with vehicle or auraptene at 30 μM for 2 h. The cell lysates were subjected to immunoblotting. (**c**) Quantification of relative band intensities of pAMPK/AMPK (*n* = 3). (* *p* < 0.05, n.s.: not significant).

As shown in Figure 2 and Figure 3, auraptene induced AMPK activation in LNCaP, PC3, and HEK-293 but not in DU145 cells. Therefore, we hypothesized that the characteristics of DU145 cells might hold the key to revealing the mechanisms underlying auraptene-induced AMPK activation. DU145 cells reportedly lack LKB1, and we confirmed this by analyzing the protein expressions of LNCaP, DU145, and PC3 cells (Figure 6a). CAMKK2 expression has been reported to be transcriptionally regulated by AR [27,28]. Furthermore, CAMKK2 expression was consistently downregulated in AR-negative cell lines, which, surprisingly, were almost undetectable in PC3 cells (Figure 6a).

Considering our earlier experimental results, we speculated that auraptene induces AMPK activation through LKB1, because auraptene does not activate AMPK in LKB1-deficient DU145 cells but activates AMPK in CAMKK2-deficient PC3 cells. We investigated whether auraptene can induce AMPK activation in DU145 cells exogenously expressing LKB1. DU145 cells were transfected with control or LKB1 plasmids for 48 h. Subsequently, the DU145 cells were treated with DMSO or auraptene at 30 μM for 2 h, and we then determined phospho-AMPK protein levels. As expected, auraptene induced significant AMPK phosphorylation in DU145 only when supplemented with LKB1 (Figure 6b,c).

Collectively, these data suggest that auraptene suppresses prostate cancer proliferation and AR activity by inducing AMPK activation, which is mediated by an increase in the cellular ADP/ATP ratio and subsequent phosphorylation by LKB1.

## 3. Discussion

We herein demonstrated that auraptene exerts inhibitory effects on both the proliferation and the migration of prostate cancer cells. Then, we showed that auraptene dose-dependently activates AMPK in LNCaP, PC3, and HEK-293 cells. Interestingly, auraptene also downregulated AR and PSA expressions in LNCaP cells. Furthermore, the inhibitory effects of auraptene on AR target gene expressions are, at least partially, reversed by siRNA-mediated AMPKα1/2 knockdown or pretreatment with the AMPK inhibitor compound C, an observation suggesting the involvement of AMPK in AR and its downstream genes, including those encoding PSA.

Several prior studies have underscored the relevance of AMPK activation and downregulation of AR target genes, though the proposed underlying mechanisms vary among reports [24,25,26]. Metformin (30 mM) reportedly decreased AR protein levels in LNCaP and C4-2B cells, with a corresponding AR mRNA decrease [24]. They also suggest that metformin-induced AR protein degradation is another layer of AMPK-mediated AR regulation [24]. A study using genome-wide expression profiling of LNCaP cells treated with AICAR or metformin revealed AR to possibly be a transcription factor downstream from AMPK [26]. That study obtained results indicating that the downregulation of AR target genes is attributable to a diminished nuclear localization of AR rather than to reductions in the mRNA or protein expressions of AR [26]. In our present experiments, auraptene decreased not only the expressions of AR target genes but also both the mRNA and the protein level of AR, which supports the former view that AMPK downregulates AR activity by decreasing AR protein levels [24]. Indeed, an AMPK-specific activator, MT 63-78, also reportedly reduced AR levels in LNCaP and C4-2 cells, and these reductions were further enhanced by co-treatment with the AR antagonist bicalutamide [25]. However, our data do not exclude the possibility of coexisting post-translational regulation of AR activity by AMPK, including its nuclear localization [26].

While androgen deprivation therapy is initially effective, prostate cancer cells eventually acquire resistance against this therapy via several mechanisms, such as amplification, transcriptional upregulation, gene mutations, and/or the generation of splice variants (such as AR-V7) of AR [29]. In castration-resistant prostate cancer (CRPC), despite reduced serum testosterone levels, AR signaling is therefore persistently activated to drive tumor progression. Currently, second-generation AR targeting agents such as enzalutamide are clinically used to combat this persistent AR activation in CRPC, though the effects are limited. Combined therapy with drugs that inhibit AR signaling at different nodes is a more effective treatment for CRPC in some cases, and AMPK-activating agents such as auraptene might be a promising option. Further studies are needed to determine whether auraptene exerts anti-cancer effects in CRPC when used either alone or in combination with available AR targeting agents.

In terms of the safety of auraptene, a previous study demonstrated that oral administration (250 mg/kg) for 28 days did not cause toxicity in rats [30], which supports its future clinical application. However, additional animal and clinical studies are needed to investigate the long-term safety of auraptene and potential drug interactions.

AMPK activation exerts an inhibitory action on energy-consuming anabolic pathways, including that of lipogenesis. AMPK blocks lipogenesis by directly phosphorylating and inhibiting acetyl-CoA carboxylase (ACC), a rate-limiting enzyme of lipogenesis, which catalyzes the reaction converting acetyl-CoA to malonyl-CoA [6], as well as by inhibiting sterol regulatory-element binding protein (SREBP) activation via several, both direct and indirect, mechanisms [31,32,33,34]. SREBP is a transcription factor known to serve as a master regulator of lipogenesis and regulates genes such as fatty acid synthase and ACC. Interestingly, androgen has been shown to promote lipogenic gene expressions by activating SREBP [35,36], and increased fatty acid synthase expressions have consistently been observed in conditions ranging from prostatic intraepithelial neoplasia to prostate cancer [37,38]. Lipogenesis is essential for rapidly dividing cancer cells that require building components for cellular membranes.

In addition to its role in lipid metabolism, mTOR/S6 kinase regulation of the cell cycle is essential for cellular proliferation. Auraptene markedly suppressed the mTOR/S6 kinase pathway, which is located downstream from AMPK. Thus, auraptene-induced AMPK activation apparently suppresses the cell growth of prostate cancer cells via at least three mechanisms, i.e., by inhibiting lipid metabolism, AR, and the mTOR/S6 kinase pathway.

Although there have been reports suggesting that AMPK may function as a tumor promoter, depending on the environment [39,40], as previously reported, auraptene effectively inhibited the proliferation of prostate cancer cells [18]. Furthermore, a very recent study confirmed genetic prostate-specific AMPK activation to significantly attenuate both the development and the progression of prostate cancer, effects attributable to the induction of PGC1α and catabolic reprogramming of prostate cancer cells, which supports the theory that AMPK activation exerts a protective effect against cancer cell proliferation [41].

In addition to suppressing prostate cancer, auraptene also reportedly suppresses the cell viability and angiogenesis of breast cancer cells by downregulating expressions of genes related to angiogenesis, such as vascular endothelial growth factor (VEGF) and VEGF Receptor 2 [42]. Another report revealed that auraptene suppresses the proliferation of breast cancer cells by inhibiting the IGF-1-stimulated S phase of the cell cycle [43]. Thus, auraptene reportedly suppresses cancer cell proliferation through multiple mechanisms.

Taking into consideration that a high concentration of auraptene suppresses the proliferation of DU145 cells, in which AMPK activation does not take place due to a lack of the LKB1 gene, it is very likely that auraptene exerts a suppressive effect on cell growth not only via an AMPK-dependent mechanism but also at least one AMPK-independent molecular mechanism. Although further study is necessary to elucidate the AMPK-independent anti-cancer mechanism(s) by which auraptene exerts its effects, the evidence that a higher concentration of auraptene is required to suppress the cellular proliferation of DU145 than that of LNCaP cells suggests the significance of an AMPK-dependent mechanism underlying auraptene-induced suppression of cell growth. 

Taken together, the data obtained in this study suggest that auraptene, a component of *Citrus hassaku*, is a potent AMPK activator. The possibility of applying auraptene to the treatment of or protection against the development of prostate cancer, including CRPC, merits further research.

## 4. Materials and Methods

### 4.1. Cell Culture

LNCaP, DU145, PC3, and HEK-293 cells were cultured in Dulbecco’s modified Eagle’s medium (DMEM) supplemented with glutamine and antibiotics (penicillin, streptomycin) and 1% fetal calf serum (FCS) under 5% CO_2_ at 37 °C. We cultured cells in medium containing 1% FCS to minimize basal AMPK activation while maintaining cell viabilities. DMEM was purchased from Nissui (Tokyo, Japan).

### 4.2. Reagents

Auraptene and dihydrotestosterone were obtained from Tokyo Chemical Industry (Tokyo, Japan), 5-aminoimidazole-4-carboxamide 1-b-D-ribofuranoside (AICAR) from Wako (Osaka, Japan), and compound C from Abcam (Cambridge, UK).

The antibodies used were purchased from Santa Cruz Biotechnology (Dallas, TX, USA) (actin: sc-47778, AR: sc-7305), Cell Signaling Technology (Danvers, MA, USA) (ACC: #3676, p-ACC: #11818, AMPKα: #5831, p-AMPKα: #2535, PSA: #5877, p70S6K: #2708, p-p70S6K: #9234, S6: #2217, p-S6: #4858, Raptor: #2280, p-Raptor: #2083), and Proteintech (Rosemont, IL, USA) (CAMKK2: #11549-1-AP).

### 4.3. ADP/ATP Assay

The ADP/ATP Ratio Assay Kit was purchased from DOJINDO (Kumamoto, Japan). LNCaP cells were seeded in a 96-well plate and incubated for 24 h, followed by administration of 30 μM auraptene (or vehicle) for 1 h. After incubation, the ADP/ATP ratio was calculated employing an ADP/ATP Ratio Assay Kit, according to the manufacturer’s instructions.

### 4.4. CCK-8 Assay

Cells were seeded in a 96-well plate and incubated for 24 h, followed by administration of auraptene (or vehicle) at the indicated concentrations and times. After incubation, 10 μL of cell counting kit-8 (CCK-8) solution (which contains the water-soluble tetrazolium dye WST-8) (DOJINDO, Kumamoto, Japan) was added to the cell culture medium, followed by incubation at 37 °C for 3 h. We subsequently measured absorbances at 450 nm in a 96-well plate. Absorbances (after background subtraction) relative to auraptene-untreated cells were used to calculate IC_50_ using ImageJ (version 1.53). 

### 4.5. Wound Healing Assay

A gap was created using the ibidi Culture-Inserts 2 Well, which provides two reservoirs for culturing cells that are separated by a 500 μm thick wall. The ibidi Culture-Inserts 2 Well was set in a 24-well plate, and 35,000 cells were seeded in each of the reservoirs and then cultured for 24 h. After monolayer formation, the ibidi Culture-Inserts 2 Well was removed, and each well was filled with medium. To evaluate cell migration ability, the gap was measured at 0 and 96 h under a microscope.

### 4.6. RNAi Interference

siRNA-mediated knockdown was performed using the reverse transfection method with Lipofectamine RNAiMAX (Invitrogen, Waltham, MA, USA), according to the manufacturer’s instructions. 2.0 × 10^5^ LNCaP cells were transfected into a 24-well plate with either 20 μM of negative siRNA (QIAGEN, Venlo, The Netherlands) or human AMPKα1/2 siRNA (Santa Cruz, sc-45312) for 72 h.

### 4.7. Real-Time Quantitative PCR

Total RNA was extracted from LNCaP cells using Sepasol reagent (Nakalai Tesque, Kyoto, Japan), and 1 μg of total RNA was used for cDNA synthesis employing Verso cDNA Synthesis Kits (Thermo Fisher Scientific, Waltham, MA, USA). Quantitative real-time PCR was performed with a CFX-96 Touch Real-Time PCR Detection System (Bio-Rad, Hercules, CA, USA) using Brilliant SYBR^®^ Green qPCR Reagents (Agilent Technology, Santa Clara, CA, USA). GAPDH served as a reference gene for normalization of AR, prostate-specific antigen (PSA), and FKBP5 expression levels. The following primers were used:

- Primers for human GAPDH: forward, 5′-GGC CTC CAA GGA GTA AGA CC-3′, reverse, 5′-AGG GGT CTA CAT GGC AAC TG-3′.
- Primers for human AR: forward, 5′-GGT GAG CAG AGT GCC CTA TC-3′, reverse, 5′-GAA GAC CTT GCA GCT TCC AC-3′.
- Primers for human PSA: forward, 5′-TCA CAG CTG CCC ACT GCA TCA-3′, reverse, 5′-AGG TCG TGG CTG GAG TCA TC-3′.
- Primers for human FKBP5: forward, 5′-AGG CTG CAA GAC TGC AGA TC-3′, reverse, 5′-CTT GCC CAT TGC TTT ATT GG-3′.

### 4.8. Immunoblot Analysis

LNCaP, DU145, PC3, and HEK-293 cells were harvested and then boiled with sample buffer containing sodium dodecyl sulfate (SDS) and 2-mercaptoethanol. Samples were subjected to SDS-polyacrylamide gel electrophoresis (SDS-PAGE) and then transferred to polyvinylidene difluoride membranes. After blocking with 3% bovine serum albumin or 3% non-fat dry milk in phosphate-buffered saline (PBS) containing 0.1% Tween20 (PBS-T) for 1 h at room temperature, the membranes were incubated with primary antibody overnight at 4 °C. After being washed three times with PBS-T buffer for 10 min, the membranes reacted with an anti-mouse or rabbit IgG horseradish peroxidase-linked secondary antibody (1:10,000) for 1 h at room temperature. After the membranes had been washed in PBS-T three times, for 10 min each time, signals were detected using Super Signal West Pico PLUS Chemiluminescent Substrate (Thermo Scientific, Waltham, MA, USA) or ImmunoStar LD (Wako). β-actin was used as the control. Band intensities were quantitatively analyzed using ImageJ (version 1.53).

### 4.9. Statistical Analysis

Statistical analyses were performed using EZR (Saitama Medical Center, Jichi Medical University, Saitama, Japan) (version 1.37) [44]. We applied the *t*-test for comparing two groups and one-way ANOVA, followed by the post hoc Dunnett’s test for multiple comparisons. Data are expressed as means ± S.E., and *p* < 0.05 was considered to indicate a statistically significant difference.

## 5. Conclusions

Auraptene activates AMPK and suppresses the mTOR/S6K pathway, as well as inhibiting the proliferation and migration of prostate cancer cells. Auraptene also downregulates AR at both the mRNA and the protein level and decreases PSA expression in LNCaP cells, actions that appear to be at least partially dependent on its AMPK activation. Auraptene-induced AMPK activation is likely to be mediated by an increase in the ADP/ATP ratio and requires LKB1. Activation of AMPK, a novel mechanism for the previously reported antitumor effects of auraptene, indicates its therapeutic potential for managing prostate cancer.

## Data Availability

The data presented in this study are available on request from the corresponding authors.

## Supplementary Materials

The following supporting information can be downloaded at https://www.mdpi.com/article/10.3390/ijms242116011/s1.
