# Supplementary material for: Auraptene Enhances AMP-Activated Protein Kinase Phosphorylation and Thereby Inhibits the Proliferation, Migration and Expression of Androgen Receptors and Prostate-Specific Antigens in Prostate Cancer Cells
Source: Int J Mol Sci. 2023 Nov 6;24(21):16011. doi: 10.3390/ijms242116011 (PMC10650886; doi:10.3390/ijms242116011)
Supplement: Supplementary file 1 [file ijms-24-16011-s001.zip › ijms-2632050-supplementary.pdf]

Supplementary Figure S1

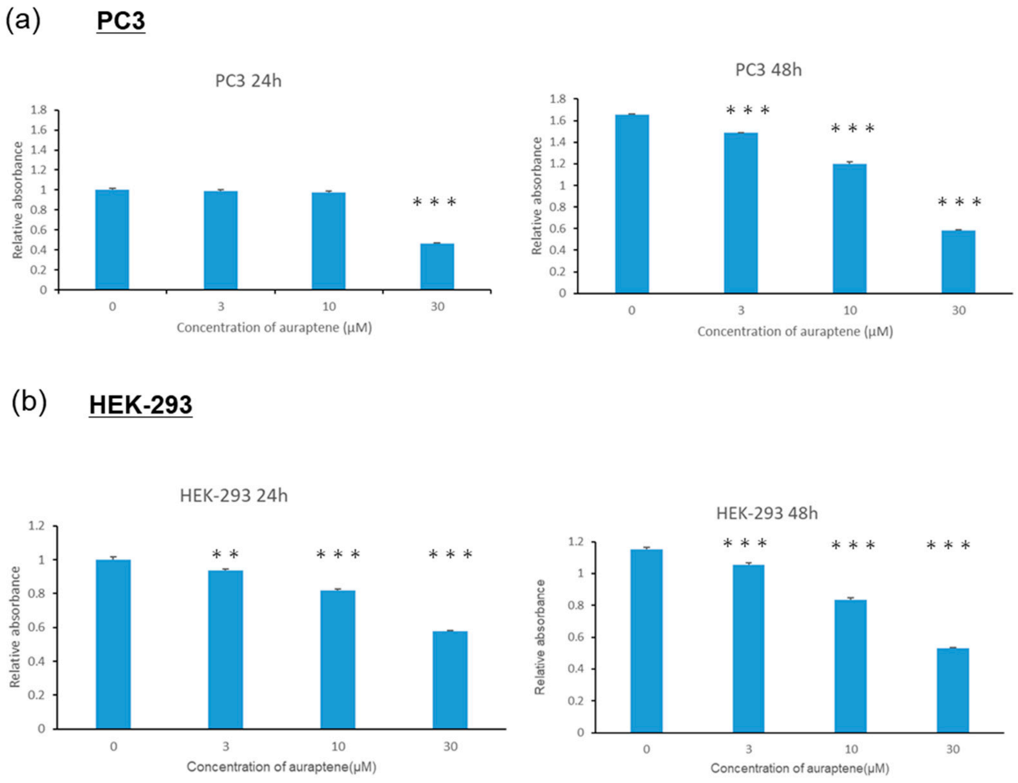

Figure S1. CCK-8 assays in PC3 and HEK-293 cells. (**a**,**b**) Relative absorbances in PC3 and HEK-293 cells. Cells were treated with auraptene (or vehicle) for 24 or 48 h at the indicated concentrations, and then absorbances at 450 nm were measured 3 h after adding CCK-8 solution. The absorbances (after background subtraction) relative to auraptene untreated cells were calculated (n = 4) (** $p < 0.01$, *** $p < 0.001$).
